# Plate waste in hospital canteens: a nutritional and environmental analysis in north-eastern Italy

**DOI:** 10.3389/fnut.2025.1542280

**Published:** 2025-05-15

**Authors:** Federica Fiori, Diana Menis, Peter Cautero, Daniela Zago, Marco Driutti, Lucia Lesa, Alessandro Conte, Enrico Scarpis, Laura Brunelli, Maria Parpinel

**Affiliations:** ^1^Department of Medicine, University of Udine, Udine, Italy; ^2^Hospital “Santa Maria della Misericordia” of Udine, Friuli Centrale Healthcare University Trust, Udine, Italy; ^3^Institute of Hygiene and Evaluative Epidemiology, Friuli Centrale Healthcare University Trust, Udine, Italy; ^4^Hospital of San Daniele-Tolmezzo, Friuli Centrale Healthcare University Trust, San Daniele, Italy; ^5^Hospital of Latisana-Palmanova, Friuli Centrale Healthcare University Trust, Palmanova, Italy; ^6^Accreditation, Quality, and Clinical Risk Unit, Friuli Centrale Healthcare University Trust, Udine, Italy

**Keywords:** waste, worksite canteen, hospital employees, lunch meal, carbon footprint, water footprint

## Abstract

**Introduction:**

Since the later a food is wasted the greater is its environmental impact, plate waste assessment is crucial to achieve a responsible consumption and production. The aim of this study was to quantify and characterize plate waste generated by users in three hospital worksite canteens in Italy.

**Methods:**

User’s trays were photographed before and after lunch consumption on 5 days. Recipes and portion sizes were provided by canteen staff. A modified Comstock scale was used to quantify plate waste. Carbon footprint, water footprint, energy and nutrient content were estimated.

**Results:**

In total, 1,227 meals were analysed. The most represented categories were females (56%) and age group 35–54 years old (49%). Plate waste ranged from 2.1% (canteen-3) to 5.9% (canteen-1). Mean plate waste was 32.0 g/tray and 38.5 kcal/tray. However, only 32% of trays contained leftovers. In this subgroup, median plate waste was 78.8 g/tray and 96.0 kcal/tray. Females wasted more than males (6.1% vs. 3.5%). The most wasted categories relative to their served amount were bread, side dishes and salad. Second courses contributed the most to the carbon and water footprint in the total sample, but in two out of three canteens the main contributor were side dishes. These were also the main contributors to the total waste in grams.

**Conclusion:**

The goal should not be only to choose a meal with low environmental impact and high nutritional quality, but also to waste less of it. Therefore, this study represents a starting point to shape tailored strategies to reduce plate waste.

## 1 Introduction

Every day our planet faces the challenge of producing enough food to feed the world’s population (1, 2). Nearly 30 percent of the world’s population is moderately or severely food insecure and almost 800 million people suffer from hunger (3). On the other hand, it is estimated that 1 billion meals a day could be produced from global household food waste, which equates to 1.3 meals/day per undernourished person (3). Throwing away edible food means wasting energy and nutrients that could be vital for others, but also has a significant impact on the environment. In order to adequately explore this topic, it is important to distinguish between food loss and food waste. The two phenomena occur at different stages of the food supply chain. Food loss includes the amount of food (for human consumption, excluding the non-edible parts) that is lost during the food supply chain before it reaches the retail level: production, post-harvest handling, storage and processing (4). On the other hand, food waste is defined as the amount of edible (4) – or edible and inedible (3) – food that is discarded from retail to the final consumer.

Food loss accounts for about 14% of global food production (4), while food waste accounts for 17% of global food production (11% is represented by household food waste, 5% by food service and 2% by retail) (5). This means that around 30% of the world’s food is thrown away uneaten, causing around 8–10% of global greenhouse gas emissions (3). For all these reasons, food loss/waste is one of the cornerstones of the 2030 Agenda, adopted by the United Nations Assembly in 2015 (Goal 12.3, which aims to halve global food waste and reduce food losses along production and supply chains) (6). According to the United Nations projections, we will reach around 10.3 billion people in the mid-2080s (7) and this means that improving our food and waste management will become increasingly important (8). Unfortunately, data on food waste in Italy (9) and the rest of the world (3) are not complete.

In food service (e.g., restaurants, work and school canteens), food waste can be further subdivided into waste generated during preparation, service, and consumption. The latter, referred to as plate waste (PW), is therefore the part of food waste for which the user is responsible and is defined as the amount of food served that is not eaten (10).

The aim of this study was to quantify plate waste generated by users in three hospital canteens in north-eastern Italy. The secondary aims were: (i) to describe the composition of plate waste in terms of energy, nutrients, environmental indicators, and food courses categories; (ii) to analyze the plate waste attitudes of users by sex and age group.

## 2 Materials and methods

The present study is part of a wider project resulting from a collaboration between the Department of Medicine of the University of Udine, the stakeholders responsible for food services, hospital management of the Friuli Centrale Healthcare University Trust, and the local network of Health Promoting Hospitals and Health Services (HPH&HS) in Udine, Italy. The aim of the entire project was to analyse the food offer and food choices to develop measures to improve nutritional and environmental awareness. The nutritional and environmental profile of the food offer and food choices in the three hospital canteens has already been reported in a previous article (11).

### 2.1 Population and setting

A convenience sampling was performed based on: geographical proximity to the University of Udine, type of management of the canteen (internal vs. external), and number of users of the canteen (high vs. low). Therefore, the study was conducted in three of the hospital canteens of the Friuli Centrale Healthcare University Trust: Udine (canteen-1, C1), Palmanova (canteen-2, C2) and San Daniele del Friuli (canteen-3, C3). C1 and C2 are managed by an external catering service but have different numbers of users per day (C1: 450–480 users/day; C2: 100–120 users/day); C3 is internally managed with 90 users/day. The target population comprised all hospitals employees who had access to the lunch service during the observed weeks (Monday to Friday). People who opted for the take-away option were not included in the analysis.

The standard composition of the tray was as follows: a first course, a second course (cold or hot), a side dish or salad, a portion of bread or two packets of crackers/breadsticks, a packet of grated cheese and a dessert including fruit. Alternatively, a second side dish can be chosen instead of the first or second course. First courses usually include pasta or rice with various sauces, soups or broths; second courses include meat (C3 did not offer beef), fish (fresh or canned) or cheese; side dishes include potatoes, cooked vegetables and pulses (pulses are also included in the first courses); desserts include fresh, canned (C3 only), or cooked fruit (C3 only), yoghurt, pudding (C1 and C2 only), and cake (C2 only). In all three canteens there is no self-service; users have to wait in a queue to receive their meals. The dishes selected by the users are served by the canteen staff in a standard portion size or in different (i.e., smaller or larger) portion sizes on request. The lunch meal has a fixed price, regardless of the composition of the lunch tray.

### 2.2 Data collection

The canteen trays of the employees who agreed to participate in the study were photographed on five consecutive working days between August and September 2022 before and after the meals were eaten. The researchers photographed the tray from above (at a 90° angle). Along with the written informed consent, participants were asked to complete a short questionnaire to collect personal data such as sex (considered as the set of biological attributes that are associated with physical and physiological features), age and typology of work (shift work/non-shift work). Participants whose questionnaires were not fully completed were excluded from the analysis. All data were pseudonymized by assigning a unique code to each participant and kept in aggregate form so that the identity of individual participants could not be traced. The study was approved by the Institutional Review Board of the University of Udine, Italy (date of approval: 06.07.2022). All study procedures complied with the ethical standards of the Declaration of Helsinki. The full description of the data collection methodology can be found in Menis et al. (11).

### 2.3 Plate waste analysis

The researchers were trained to identify the standard portion for each food item by showing them a sample of photographs. The categories of food were: first course, second course, side dish, salad, hard grating cheese, bread or cracker, dessert. The composition of the tray was estimated through a blind analysis of the photographs to identify the recipes, estimate the portions (e.g., 50, 100, 150%) compared to the standard one (100%) and to estimate percentage of PW (edible part) using a modified Comstock scale (12). The scale was modified by considering quarters of the standard portion and taking into account the fact that if the standard portion remained completely in the tray after the meal, this would correspond to a 100% PW. Wasting half of a portion twice size would be equivalent to wasting a full standard portion (i.e., PW = 100%). Then dividing the percentage of visually estimated PW by the percentage of the chosen portion size, gives the final variable PW (%) (Table 1).

**Table 1:** Definition of the plate waste variables and their full description.

Acronym	Definition	Description
PW (%)	Plate waste (visually estimated)	Edible part discharged / Edible part placed on the tray *100
PW (g)	Plate waste in grams	Grams of discharged food
PWE (%)	Plate waste in percentage of energy	Energy discharged / Energy placed on the tray *100
PWE (kcal)	Plate waste in energy	Energy content of discharged food

Considering that photographs and PW (%) are not sufficient to obtain a complete dietary assessment, they need to be supported by additional food composition data (13). Therefore, the recipes and portion sizes for each dish offered during the observation period were requested from the canteen staff. This information was matched at an ingredient-level with the foods of the Italian Food Composition Database for Epidemiological Studies (BDA) version 2022 (14) and with those of the SU-EATABLE LIFE dataset (15) to obtain the energy and nutrient profile as well as the environmental indicators (i.e., carbon footprint – CF; water footprint – WF) for each standard portion of the final dish. This process was performed in accordance to the standard recipe approach (16). However, due to lack of data, we did not consider in the environmental analysis the post market phases of the life cycle of each food, such as the cooking phase, and (in C1 and C2) the transport from the cooking centre to the canteen that occurred for some recipes.

As a result, PW (g) was estimated based on PW (%) and the grams of the standard portion. Similarly, PWE (kcal), nutrient composition, CF and WF of PW were estimated based on the data from the previously described databases and portion sizes. Finally, the percentage PW in terms of energy content (PWE, %) was calculated by dividing the energy content of the wasted food by the total energy content of the chosen food.

### 2.4 Statistical analysis

A descriptive analysis of participants’ characteristics associated with each tray was performed, grouped by fully or not fully consumed meals and by canteen. Descriptive analyses were performed on the following variables by canteen and in the total sample: PW (g, %), PWE (kcal, %), PW composition in terms of nutrients (proteins and lipids –total, animal and vegetal– available and soluble carbohydrates, fiber, saturated, monounsaturated and polyunsaturated fatty acids), CF and WF. The PW (%) was calculated for each food category and for the entire meal. Additionally, the estimated weight of PW in grams was used to calculate the percentage by weight of each food course category relative to the total weight of PW in each canteen. The graphical representation of this percentage should provide insight into which category was wasted the most during the 5 days of analysis, disregarding of the served amount. Data were presented as *N* (%) or median (25th-75th percentile) as most variables were not normally distributed according to the Shapiro–Wilk test. The median values of all PW variables were then calculated for the subgroup of trays with leftovers (meals not fully consumed).

Additional analyses were performed excluding canteen accesses subsequent to the first access by the same users in the same week (each participant was considered only once). The aim of this was to examine the PW based on the actual characteristics of the users: sex (male, female) and age group (I, <34 years; II, 35–54 years; III, >55 years). The Kruskal-Wallis test was applied to determine whether the median scores of the PW variables considered (PW in grams, PWE in kcal, protein, lipids, carbohydrates, fiber, CF and WF) differed by age group (I, II, and III) in the total sample (C1 + C2 + C3) and among canteens. If the Kruskal-Wallis test was significant, the Dunn test was performed as a post-hoc analysis to determine which age group differed. In addition, the same PW variables were compared between females and males using the Wilcoxon rank sum test. *p*-values <0.05 were considered statistically significant. Data were analysed using SAS Enterprise Guide version 7.15 (2017. SAS Institute INC., Cary, NC, USA).

## 3 Results

### 3.1 Description of the study sample

A total of 1,227 lunch meals were analysed, of which 798 belong to C1, 228 to C2 and 201 to C3. Based on the mean daily number of users of each canteen, we estimated a response rate of 34% in C1, 40% in C2 and 45% in C3 (12). Females were 56% of the total sample and the mean age of participants was lower in C1 (43 ± 12 years) and C2 (44 ± 12 years) then in C3 (49 ± 11 years). The majority of the sample were non-shift workers in C1 (69%), C2 (71%), and C3 (80%). The full description of the characteristics of participants associated to the analysed trays and the nutritional composition of their food choices can be found in a previous publication (12). In the total sample, 32% of the trays contained leftovers (i.e., not fully consumed meals). A higher percentage of not fully consumed meals was observed in C1 (N = 300, 38%) than in C2 (*N* = 52, 23%) and C3 (*N* = 43, 21%). As can be seen in Table 2, females and males show similar distribution when examining the subgroups of fully and not fully consumed meals. Percentages of fully consumed meals increased from age group I to age group III in C1 and C3.

**Table 2:** Characteristics of participants associated to the total sample of analysed trays (*N* = 1,227), grouped by canteen and fully/not fully consumed meals.

Category	Variable	C1			C2			C3		
		FCM (*N* = 498)	NCM (*N* = 300)	Total (*N* = 798)	FCM (*N* = 176)	NCM (*N* = 52)	Total (*N* = 228)	FCM (*N* = 158)	NCM (*N* = 43)	Total (*N* = 201)
Age group, *N* (%)	I (≤ 34)	120 (50)	119 (50)	239	55 (77)	16 (23)	71	16 (62)	10 (38)	26
	II (35–54)	248 (65)	134 (35)	382	84 (79)	23 (21)	107	85 (78)	24 (22)	109
	III (≥ 55)	130 (73)	47 (27)	177	37 (74)	13 (26)	50	57 (86)	9 (14)	66
Sex, *N* (%)	Female	248 (62)	154 (38)	402	116 (77)	34 (23)	150	110 (79)	30 (21)	140
	Male	250 (63)	146 (37)	396	60 (77)	18 (23)	78	48 (79)	13 (21)	61
Typology of work, *N* (%)	Shift worker	116 (57)	86 (43)	202	36 (73)	13 (27)	49	27 (75)	9 (25)	36
	Non-shift worker	357 (65)	195 (35)	552	126 (77)	37 (23)	163	129 (80)	32 (20)	161
	Missing	25 (57)	19 (43)	44	14 (88)	2 (13)	16	2 (50)	2 (50)	4

Data are expressed in absolute number and row percentage in parenthesis; C1, canteen-1; C2, canteen-2; C3, canteen-3. FCM, fully consumed meals; NCM, not fully consumed meals.

### 3.2 Plate waste and its nutritional and environmental impact

The mean value of plate waste across all trays analysed (fully consumed and not fully consumed) was 32.0 g/tray and 38.5 kcal/tray, corresponding to 61.3 g CO_2_ eq., and 53.7 L H_2_O (Supplementary Table 1). However, the median value was zero, meaning that more than half of the trays in each canteen were waste-free. Table 3 presents data for the subgroup of meals not fully consumed, whose median PW was 78.8 g and 96.0 kcal. Supplementary Figure 1 shows the difference between the three canteens in terms of PW (g) (*p* = 0.0010), with C1 and C2 significantly different from C3 (*p* ≤ 0.0083).

**Table 3:** Plate waste from meals not fully consumed and its composition (energy and macronutrients), CF, and WF by canteen and in the total sample (*N* = 395).

Variables	C1 (*N* = 300)	C2 (*N* = 52)	C3 (*N* = 43)	Total (*N* = 395)	*p*-value
	Median (25th-75th percentile)				
PW (g/tray)	79.4 (46.8–150.0)	89.9 (46.4–135.6)	42.5 (35.4–85.0)	78.8 (42.5–141.9)	0.0010*
PWE (kcal/tray)	99.6 (45.7–170.7)	100.4 (57.7–162.0)	50.8 (25.4–105.4)	96.0 (44.3–162.1)	0.0008*
Total protein (g/tray)	3.6 (1.4–8.9)	3.3 (1.8–5.3)	1.1 (0.6–3.0)	3.2 (1.3–7.2)	<0.0001*
Animal protein (g/tray)	0.0 (0.0–5.2)	0.0 (0.0–1.0)	0.0 (0.0–0.1)	0.0 (0.0–3.8)	0.0371*
Vegetal protein (g/tray)	1.9 (0.9–4.1)	2.5 (1.1–3.9)	1.1 (0.6–2.6)	1.9 (0.7–3.7)	0.0038*
Total lipids (g/tray)	3.1 (1.2–7.0)	4.2 (2.4–7.4)	2.3 (0.6–3.7)	3.1 (1.7–7.0)	0.0596
Animal lipids (g/tray)	0.0 (0.0–1.9)	0.0 (0.0–1.4)	0.0 (0.0–0.5)	0.0 (0.0–1.8)	0.2024
Vegetal lipids (g/tray)	2.0 (0.2–4.5)	2.6 (0.2–6.1)	1.9 (0.2–3.7)	2.1 (0.23–4.8)	0.2948
Available carbohydrates (g/tray)	10.4 (2.8–24.7)	10.5 (3.8–17.8)	2.6 (1.3–13.7)	9.0 (2.7–20.7)	0.0022*
Soluble carbohydrates (g/tray)	1.6 (0.7–3.8)	1.4 (0.5–3.5)	1.3 (0.4–2.6)	1.5 (0.6–3.8)	0.3324
Fiber (g/tray)	1.5 (0.8–2.7)	1.3 (0.7–2.6)	0.8 (0.7–1.8)	1.4 (0.7–2.6)	0.0709
Saturated fatty acids (g/tray)	0.54 (0.3–1.2)	0.85 (0.41–1.53)	0.35 (0.28–0.7)	0.55 (0.26–1.23)	0.0455*
Monounsaturated fatty acids (g/tray)	1.33 (0.4–3.6)	1.80 (0.94–4.09)	1.31 (0.18–2.6)	1.34 (0.55–3.48)	0.2159
Polyunsaturated fatty acids (g/tray)	0.41 (0.16–1.2)	0.49 (0.24–0.97)	0.18 (0.08–0.6)	0.39 (0.16–1.0)	0.0207*
CF (g CO_2_ eq. /tray)	104.5 (44.1–245.2)	82.3 (34.8–130.4)	34.2 (19.5–67.7)	87.6 (33.7–239.2)	<0.0001*
WF (L H_2_O/tray)	112.7 (51.6–234.5)	110.7 (54.3–161.8)	53.6 (34.7–74.9)	100.7 (50.0–192.5)	<0.0001*

C1, canteen-1; C2, canteen-2; C3, canteen-3; PW, plate waste; PWE, plate waste in terms of energy CF, carbon footprint; WF, water footprint. * *p* < 0.05.

The PW (Table 3) was composed in median by 3.2 g protein (mainly vegetal), 3.1 g lipids (mainly vegetal), 9.0 g available carbohydrates, 1.4 g fiber, and 0.39 g polyunsaturated fatty acids. Looking at the amount of nutrients served in the total sample, the most wasted nutrients were fiber (5.9%), vegetal proteins (5.6%) and vegetal lipids (5.5%). The other nutrients ranged from 3.2% (animal fats) to 4.8% (polyunsaturated fatty acids).

CF and WF of PW amounted to 87.6 g CO_2_ eq. and 100.7 L H_2_O per tray. The median CF and WF values of the plate waste were higher in C1 (104.5 g CO_2_ eq.; 112.7 L H_2_O) than in C2 (82.3 g CO_2_ eq.; 110.7 L H_2_O) and C3 (34.2 g CO_2_ eq.; 53.6 L H_2_O) (*p* < 0.001).

### 3.3 Plate waste and its food course categories

**Figure 1:**
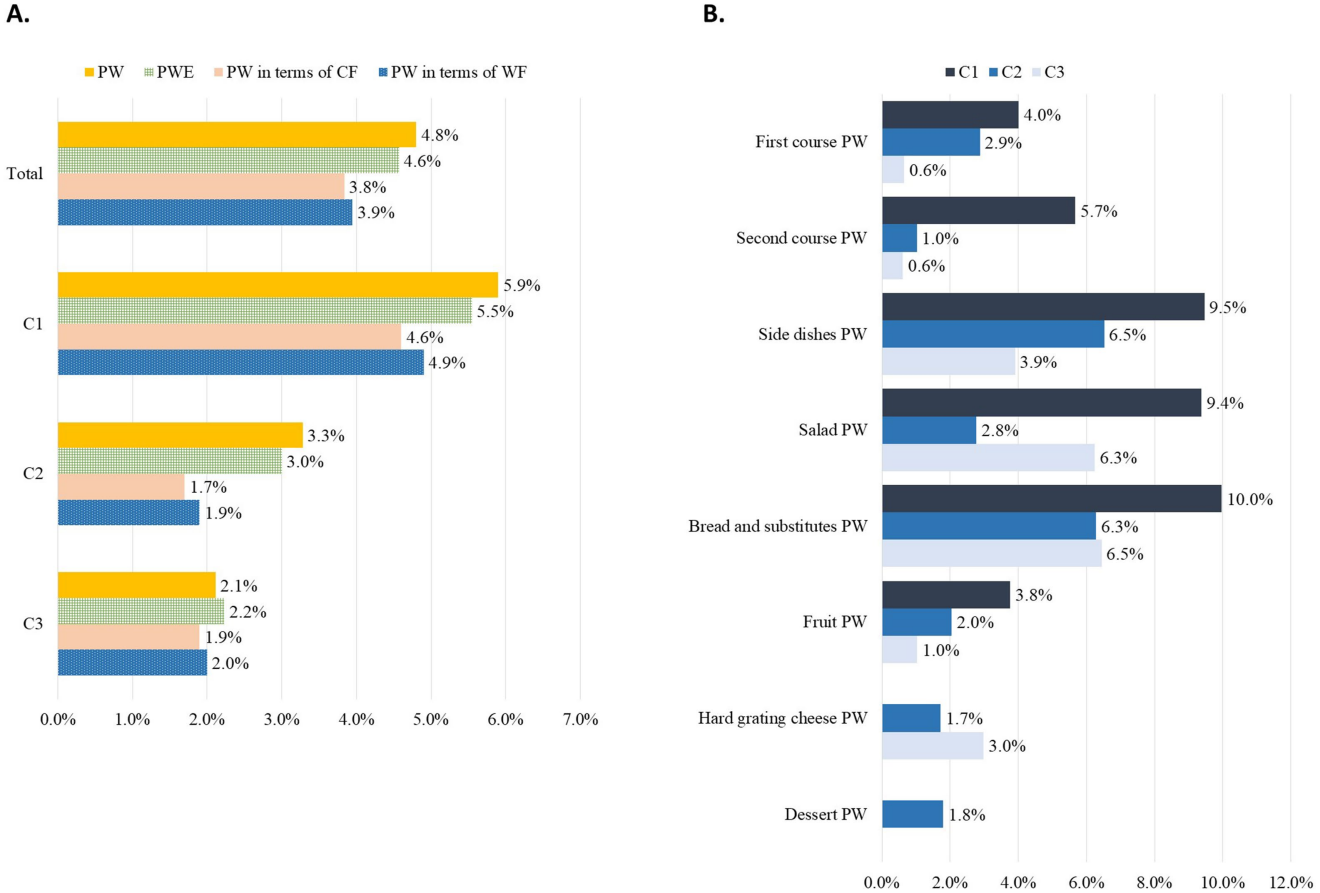
Percentage plate waste (weight, energy, CF and WF) for the entire meal **(A)** and PW (%) by category **(B)**, calculated for each canteen (*N* = 1,227).

Considering all three canteens together, the PW was 4.8%, but the highest percentage of PW (%) was found in C1 (5.9%) (Figure 1A). Accordingly, the wasted percentage of energy (i.e., PWE) CF, and WF over the served amount were higher in C1 (5.5, 4.6, and 4.9%, respectively) than in C2 (3.0, 1.7, and 1.9%, respectively) and C3 (2.2, 1.9, and 2.0%, respectively). When analysing each food course category (Figure 1B), C1 had the highest percentages in all categories, with the exception of hard grating cheese and desserts, which did not generate any waste. Desserts were fully consumed also in C3. The least wasted food course category in C2 was the second course. The “bread and substitutes” category was the most wasted in C1 (10% of the served category) and in C3 (6.5% of the served category). In C2, side dishes were the most wasted food course category, accounting for 6.5% of served food.

**Figure 2:**
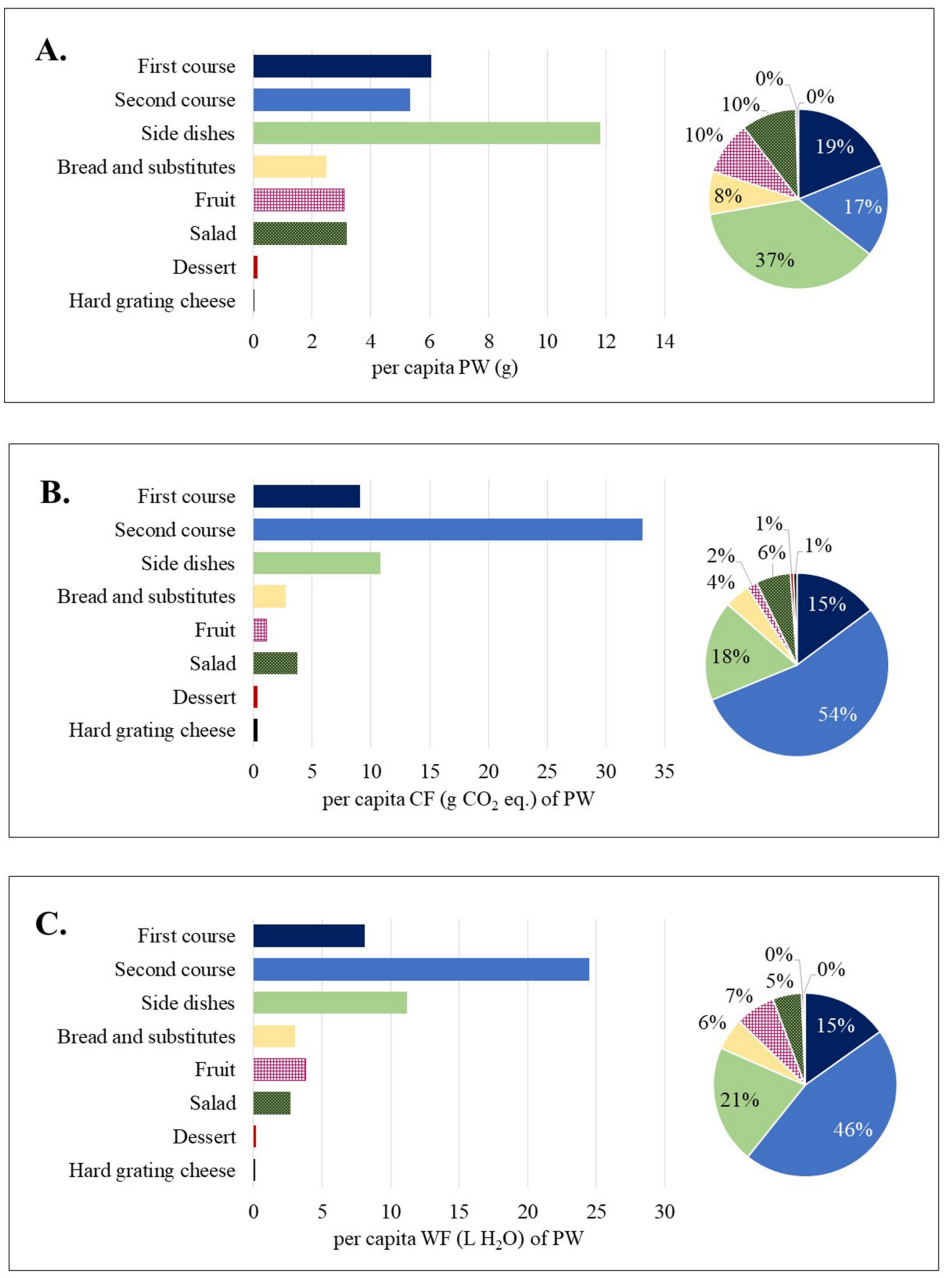
Per capita plate waste and percentage contribution to the total amount of plate waste by food course category [**(A)**, weight; **(B)** carbon footprint; **(C)** water footprint] in all canteens.

Looking at the total amount of PW (g) produced by all the analysed trays, side dishes were the main wasted food category, accounting for 37% of total PW (Figure 2A), which corresponds to an average value of 11.8 g/tray in the total sample (Supplementary Table 2A). Even when looking at the individual canteen separately, the main wasted category was side dishes. However, we observed differences by canteens in the second most wasted food course category (Supplementary Table 2A). Specifically, in C1, first and second courses accounted for 18 and 20%, respectively, of the total amount of PW; in C2, first courses accounted for 27% of the total amount of PW; in C3, salad accounted for 28%. In the total sample, second courses contributed the most to the CF and WF of PW (54 and 46%, respectively; Figures 2B,C) and the same was in C1 (58 and 50%, respectively; Supplementary Tables 2B,C). In contrast, in C2 (CF: 29%, WF: 34%) and C3 (CF:32%, WF:35%), side dishes contributed the most to the environmental indicators of PW.

### 3.4 Plate waste behavior by participant characteristics

**Figure 3:**
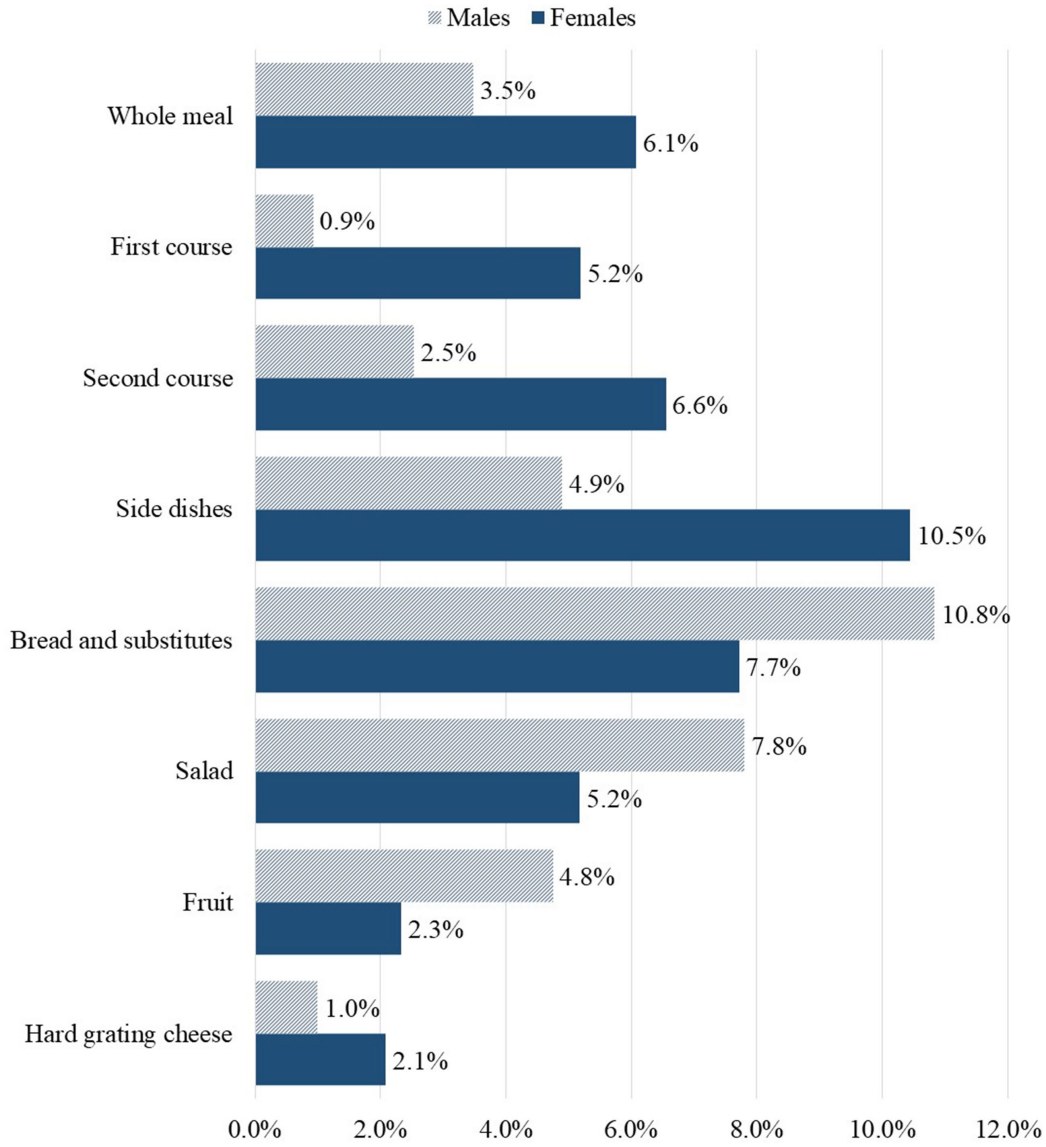
Percentage of plate waste for the whole meal and by category calculated in females (*N* = 322 of which 109 with a not fully consumed meal) and males (*N* = 236 of which 75 with a not fully consumed meal) considering their first canteen access.

Supplementary Table 3 shows the characteristics of PW, stratified by age group and sex. Regarding the age group, although there is a trend toward higher median values for all variables in the youngest group compared to the others, the only difference was found in the WF of PW. In this case, the median value was significantly higher in the youngest group than in the oldest group (Dunn test, *p* = 0.0343). The median value of PW for females was significantly higher than those for males in terms of grams (84.6 g in females vs. 50.0 g in males), energy, protein content, lipid content, CF and WF. Females percentage PW (6.1%) was also higher compared to males (3.5%), as shown in Figure 3. Specifically, females wasted more first courses (+4.3%), second courses (+4.1%), and side dishes (+5.6%) than males relative to the served amount.

## 4 Discussion

This study provides data on the PW produced by the users of worksite canteens in three hospitals in north-eastern Italy. To our knowledge, this is the first study to estimate the amount and characteristics of PW in an Italian workplace canteen. We found that most trays did not contain PW. An analysis by sex revealed that females produce more PW than males. Overall, our PW ranged from 2.1% (C3) to 5.9% (C1) in the total sample, with a mean of 32.0 ± 63.9 g/tray and a median value, when trays without leftovers are excluded, ranging from 42.5 g (C3) to 89.9 g (C2) per meal. The median CF and WF values of PW ranged from 34.2 g CO_2_ eq. /tray (C3) to 104.5 g CO_2_ eq. /tray (C1) and from 53.6 L H_2_O /tray (C3) to 112.7 L H_2_O /tray (C1). Similar to what observed in a previous study in Chinese working buffet-style canteens, C3 which offers less cuisine diversity, and was self-managed, produced less per capita PW than C1 and C2 (17).

### 4.1 Plate waste quantification

The percentage of trays without PW that we found (62–79%) is comparable to that found in a German university canteen with selectable pre-portioned served food (except salad, which was offered as a buffet) (75%) (18) and in a survey of plate clearing habits among university students in UK (78%) (19). Nonetheless, as expected, our percentage of trays without PW was way higher than the one found at a pre-paid all you can eat university dining facility in the USA (39%) (20).

Indeed, the available data in the literature on PW are highly heterogenous (21, 22), depending on the data collection method, the type of food service and setting, the users population, the type of distribution (e.g., self-service buffet or served), the type of menu (e.g., availability of food options or fixed menu) and the price (e.g., fixed, by dish, or by weight). For example, Roe et al. reported a low percentage of daily PW (3.3%) when they analysed free-living conditions (all-day meals, not exclusively canteens) and a very high PW (39%, 203 g per capita) in a laboratory setting where participants selected their meal from a limited offering (22). The only studies that actually analysed a hospital workplace canteen which is similar to our setting were conducted in Brazil (23, 24) and in Denmark (25), although they differ from our setting due to serving type (i.e., buffet or partially served), the way meals are paid for (i.e., fixed price menu or buffet with payment by weight), as well as potential socio-cultural differences and sensitivity to the problem of food insecurity. Beside these differences, the Brazilian studies (6–7.7%; 20–37 g) (23, 24) and the Danish study (4.5–5.4%; 17–25 g) (25) found PW values similar to ours. The results from other studies, which were conducted in European worksite canteens, showed heterogeneous results with PW ranging from 21 g to 108 g per meal with a buffet (26, 27) or pre-portioned dishes (28). In university settings, the reported PW ranged from 62 to 200 g per meal, including US universities where service was provided in the form of prepaid all you can eat buffet (20, 29), a Portuguese university where there was a fixed self-service menu (30), and Chinese universities (31, 32), where the dinning system works like a shop with convenient pricing (31).

### 4.2 Characterization of plate waste and its nutritional and environmental impact

Regarding the nutritional impact of PW, we found that in the total sample of trays, a mean of 38.5 kcal per meal per capita is wasted. PWE ranged from 2.2% in C3 to 5.5% in C1. No data were found in the literature on the nutritional composition of PW in adult catering facilities. PWE reported for school children in Italy is generally high, accounting for 26 and 36% in two case studies, with fiber being one of the most wasted nutrients (33). Despite the differences in the population of analysis, food service characteristics (i.e., fixed menu in schools), and amount of PW, our results similarly show that fiber was the most discharged nutrient when considering the wasted/served ratio.

The great relevance of the PW assessment lies in the fact that the later a food is wasted in the supply chain, the greater its environmental impact, considering the higher investment in terms of processing, transportation, cooking and consequently in terms of greenhouse gas emissions (34). In the present study CF of the wasted food represent the 3.8% of the emissions of the total served food and wasted WF represent the 3.9% of the WF of the served food, corresponding to an average of 61.3 g CO_2_ eq./tray and 53.7 L H_2_O in the total sample of trays analysed. Therefore, we can estimate that considering all the served meals of the three canteens (about 3,350 meals a week) (11), the WF and CF emissions would be of 205.4 kg CO_2_ eq. and 179,895 L H_2_O each week.

Previous studies in Chinese university canteens found an average PW of 258.6 g CO_2_ eq. per meal in a shop type dining facility (not exclusively lunch) (31), which is higher than our mean value considering the total sample (61.3 ± 197.1 g CO_2_ eq./tray), and a daily per capita CF of plate waste ranging from 77 to 450 g CO_2_ eq. (32). In worksite buffet-type canteens in China (17), average lunch CF and WF of PW were about 900 g CO_2_ eq. and 250 L per capita (vs. 53.7 ± 142.7 L H_2_O in our sample). However, variability is high depending on type of wasted foods. Indeed, it is known that meat and cheese, typically served as second courses in Italy, have the greatest environmental impact (15). In the present study, the per capita PW in terms of CF and WF was five times higher in C1 than in C3, while the PW in grams was three times higher than in C3. These differences let us suppose that different typologies of food have been wasted in the canteens, with more second course waste in C1, which was confirmed in our results. Moreover, in C1 second courses were the main contributors to the total CF and WF, similarly to what observed by previous authors (17, 35). Although we obtained similar results when considering the three canteens together, the lower contribution of second courses to the CF and WF of PW observed in C2 and C3 could indeed be explained by the lower offer and choice of beef in C2 and the absence of beef offer in C3 (11). Indeed, in C2 and C3, side dishes contributed the most to the CF and WF of PW. Despite the lower environmental impact of plant-based foods compared to animal-based foods (15), in these two canteens, the large amount of wasted side dishes justifies that this category is the main contributor to the environmental indicators. Indeed, side dishes (in our study represented by vegetables, potatoes and pulses) contributed the most to the total weight of PW when combining the data from the three canteens. This confirms that the PW resulting from our analysis consisted mainly of carbohydrates and vegetal proteins and fats.

The characterization of PW in terms of the total amount of wasted food categories shows that after side dishes (36.8%), first and second courses accounted for the largest proportion of total PW (18.8 and 16.6%, respectively). In a previous whole day analysis by Partearroyo et al. (36), which included both home and non-home PW, the most wasted foods were bread (25% of total PW), main courses (16%), and first and second courses (15% each). Bread was not one of the main contributors to the total PW in our study, but it was the category most wasted in C3 and C1 in relation to the amount served, suggesting that the low weight of wasted bread is related to the low amount served. In a previous study conducted in Italian schools (33), the most wasted categories were fruit, bread, and side dishes when considering the percentages of the amount served, which partially overlaps with our results (except for fruit, which was not highly wasted in our adult sample). When analysing PW in relation to the amount served, in our study side dishes were the most wasted category in C2, the second most wasted category (after bread and substitutes) in C1 and the third most wasted (after bread and substitutes, and salad) in C3. Previous data highlighted that the higher the quality of the diet (according to the Healthy Eating Index-2015), the higher the amount of food wasted (37). Therefore, the goal should not only be to choose a meal that is richer in plant-based products, but also to waste less of the plant-based foods.

### 4.3 Plate waste and demographic characteristics

In contrast to what is reported in the literature (38), we found the same distribution of wasters among females and males. However, our results confirm the data in the literature that females produce more weight of PW than males (24, 28, 31, 35, 36, 39). We also found more PWE, proteins, lipids, CF and WF of PW in females than in males, which in our case seems to derive from the higher amount of PW produced by females in all food course categories except bread and substitutes, salad and fruit. To our knowledge, only one study did not find difference by sex when analysing the weight of PW, even considering each food category (40). The different behaviors observed in females and males could be related to the offer of standard portions in the canteens considered, which prevents the possibility of adapting portions to energy needs, which are generally higher in males than in females (41). This hypothesis about overly generous portions is in line with the reports of other authors (28, 39). Aligned to this hypothesis, gender differences were not found under free-living conditions, but only in laboratory-based meals where portion sizes were predetermined and the same for females and males (22). One strategy to reduce PW could therefore be to reduce portion sizes (42) or to reduce the size of the plate in order to disguise a smaller portion (43). However, in a recent study (24), women wasted more than men, even when they chose a smaller portion (−34%) in a buffet food service. Therefore, portion oversizing could be only one of the many factors influencing the differences in PW between females and males. As an example, in a previous study (44) men were found to be more conscious of food waste than women, and gender stereotypes on women (i.e., eating smaller amounts of food rated as more feminine) may influence their attitude toward PW (28).

In terms of age groups, we found more plate cleaners (those who fully consumed their meal) in the older age group (age group III, ≥ 55 years old) compared to younger age groups in two out of three canteens. This was also confirmed by a trend towards a decreasing amount of PW from age group I (≤ 34 years old) to age groups II and III. We can hypothesize that there may be a difference in mindset that favours wasting in the younger generations for cultural reasons. Previous data has shown that food insecurity possibly experienced in the past and emotional reasons (guilt and empathy) are the main determinants of sustainable food waste practices in older people (45). However, findings on this topic are mixed in the literature, with some authors observing no association between age and PW (28, 39) and others reporting a similar trend to the one we found, with lower PW among older age groups (≥65 years old) compared to younger adults in household and free-living conditions settings (36, 46). However, the main source of these data come from households (36, 46), where food management is a key determinant of food waste production, or from self-reported data from the food service setting (28, 39), which may not represent the actual PW. Indeed, Sebbane and Costa (28) reported a discrepancy between self-reported and observed PW behavior.

### 4.4 Implications and recommendations for the future

The United States Environmental Protection Agency (EPA) in 2023 developed the Wasted Food Scale to show which are the actions that need to be prioritized to avoid the food waste in an optic of sustainability and circular economy (47). The best practice, that is also the one that offer more benefits, is to prevent the wasting of food. Following this principle, our suggestions to reduce PW are to increase the awareness about the nutritional and environmental impact of PW with specific interventions and practical aids, reduce portion sizes or make them more flexible, ensure a palatable food offer, and give financial incentives in case of zero plate waste or implement pricing by weight. As an example, it has been observed that restaurants with different price charging according to the amount of food have an average plate waste smaller than fixed price table service (48). The reduction of PW would contribute to the achievement of the SDG 12 (responsible consumption and production), but also indirectly of the SDG 2 (zero hunger), and 3 (good health and well-being).

For an overview of the actions that can be implemented, it is necessary to calculate the total food waste in the canteen, from food distribution to food consumption. Therefore, the next step of our ongoing project, will be to estimate how much food is left on the counters of the canteen at the end of the service, and how the waste is handled. Indeed, at least a part of the food that the canteen does not distribute can be used to create new recipes offered during the next lunch (upcycle – second option of the scale) or can be donated to be used as food (second option of the scale) or as resource to be recovered since it contains useful nutrients (feed or anaerobic digestion– last options of the scale). Indeed, the results of the present study in terms of characterization of the plate waste energy and nutrient composition could also be important to inform better food waste management by addressing it to the most efficient disposal. As an example, food waste could be transformed in valuable resources such as biogas, biochar, or compost (49).

### 4.5 Strengths and limitations

The results of this study should be considered in light of some limitations. The first limitation arises from the questionnaire, which did not include questions on, for example, perceptions of food quality/palatability, adequacy of portion size, anthropometric measures and attitudes towards environmental issues. Moreover, our analysis focused on the midday meal and the questionnaire did not include information on users’ overall food consumption during the day. We chose to minimize the length of the questionnaire to make it easier for users to participate by reducing the completion time during their lunch break. Second, we cannot exclude the occurrence of selection bias, due to limited lunch break time discouraging users to participate, and observation bias, due to the presence of researchers in the canteen. However, by reducing at minimum the effort required to user, we reached a sample of about half the served trays, leading to a sample that can be considered representative of habitual users of the three canteens, but not representative of all hospital employees. Regarding the observation bias, the presence of the researchers at the canteen was not invasive: at the end of the lunch break, users independently placed their coded trays in the collection racks, and then the researcher photographed them. Third, we did not consider total waste generated in the canteens (i.e., food waste generated at the serving point). Fourth, the SU-EATABLE LIFE (SEL) dataset was used to estimate CF and WF. However, it only includes data up to the market phase. This may have generated an underestimation of CF and WF in cooked foods both due to the impact of cooking and additional transportation from cooking centre to the canteen (in C1 and C2). Finally, the generalizability of the results could be limited by the different food offerings in each canteen, which influence user behavior, and due to the peculiarities in terms of traditional composition of the meal of different countries that limits comparison at an international level.

Nonetheless, the main strengths of the present study are: (1) the provision of initial data on PW in Italian food service setting, which is lacking in the literature, and (2) the analysis of PW from different points of view (energy and nutrients, environmental indicators, food course categories). In addition, the PW was not self-reported but visually estimated in a more objective way using digital photos taken by the researchers before and after the consumption of each meal. Indeed, questionnaires compiled by the users were previously seen to underreport PW (28). Although less accurate than direct weight measurement (gold standard), weight estimates based on photos require minimal disturbance to the eating environment and correlate with actual food weight (50). The accuracy of visual estimates was further enhanced in our study by the fact that by the canteen staff provided all recipes along with their standard portion sizes. Furthermore, using the digital photo method instead of aggregate weight measurements allowed us to estimate individual (i.e., per tray) PW and to analyse some potential demographic determinants of PW such as sex and age.

## 5 Conclusion

The present study shows a relatively low amount of plate waste in three hospital workplace canteens in Italy compared to international literature data. However, to our knowledge, there is no established threshold for PW that can be generally considered acceptable; PW should be limited as much as possible. In our case, the PW consisted mainly of side dishes, while the most wasted food course categories relative to their served amount were bread, side dishes and salad. Regarding the environmental impact of the PW, in the total sample the second courses contributed the most, but in two out of three canteens the main contributor was the side dishes category. Finally, the analysis by sex showed that PW was higher in females than in males.

Although the data on PW in foodservice cannot be easily generalized, it could provide a starting point for the development of strategies and measures to reduce PW. Based on our findings, interventions should focus on: (1) tailoring portion size with the possibility to choose smaller or bigger portion based on individual needs (e.g., gender differences); (2) raising awareness of the environmental impact of PW and the importance of the small individual contribution to achieve a bigger goal; (3) educating not only about choosing more sustainable dishes (e.g., plant-based foods) but also about wasting less of them. Indeed, even if they have a low CF and WF, they can lead to significant environmental impacts when all users are taken into account.

Further studies are needed to fully characterize food service waste from the kitchen to the customer, taking into account not only the consumption phase but also the preparation and service phases. Quantifying each stage of food waste production could be useful for targeting actions to the stages with the greatest impact on food supply in the workplace.

## Data Availability

The raw data supporting the conclusions of this article will be made available by the authors, without undue reservation.
