# Supplementary material for: Plate waste in hospital canteens: a nutritional and environmental analysis in north-eastern Italy
Source: Front Nutr. 2025 May 15;12:1542280. doi: 10.3389/fnut.2025.1542280 (PMC12119306; doi:10.3389/fnut.2025.1542280)
Supplement: Supplementary file 1 [file Data_Sheet_1.DOCX]

Supplementary Material

***Plate waste in hospital canteens: a nutritional and environmental analysis in north-eastern Italy***

**Supplementary Table 1.** Plate waste and its composition (energy and macronutrients), carbon footprint, and water footprint considering the total sample of analysed trays (N=1227).

|  | **Mean** | **SD** | **Median** | **IQR** |
| --- | --- | --- | --- | --- |
| PW (g/tray) | 32.0 | 63.9 | 0.0 | 0.0-40.0 |
| PWE (kcal/tray) | 38.5 | 81.0 | 0.0 | 0.0-37.7 |
| CF (g CO_2_ eq./tray) | 61.3 | 197.1 | 0.0 | 0.0-31.7 |
| WF (L H_2_O/tray) | 53.7 | 142.7 | 0.0 | 0.0-38.7 |

Notes: PW, plate waste; PWE, plate waste in terms of energy CF, carbon footprint; WF, water footprint; SD, standard deviation; IQR, interquartile range.

**Supplementary Figure 1.** Plate waste (g) of not fully consumed meals by canteen (C1, canteen 1; C2, canteen 2; C3, canteen 3) (N=395).

P=0.0083*

P=0.0008*

**Supplementary Table 2.** Grams (**A**), carbon footprint (**B**) and water footprint (**C**) of plate waste by course category and each percentage contribution (%) to the total amount of plate waste in each canteen and total.

| **A.** | **C1** | | | **C2** | | | **C3** | | | **Total** | | |
| --- | --- | --- | --- | --- | --- | --- | --- | --- | --- | --- | --- | --- |
| **Food course category** | **total PW (g)** | **per capita PW (g)** | **Contrib (%)** | **total PW (g)** | **per capita PW (g)** | **Contrib(%)** | **total PW (g)** | **per capita PW (g)** | **Contrib(%)** | **total PW (g)** | **per capita PW (g)** | **Contrib(%)** |
| First course | 5799 | 7.3 | 18% | 1361 | 6.0 | 27% | 250 | 1.2 | 10% | 7410 | 6.0 | 19% |
| Second course | 6187 | 7.8 | 20% | 250 | 1.1 | 5% | 97 | 0.5 | 4% | 6533 | 5.3 | 17% |
| Side dishes | 11337 | 14.2 | 36% | 2135 | 9.4 | 42% | 1002 | 5.0 | 39% | 14473 | 11.8 | 37% |
| Bread and substitutes | 2506 | 3.1 | 8% | 300 | 1.3 | 6% | 250 | 1.2 | 10% | 3056 | 2.5 | 8% |
| Fruit | 3168 | 4.0 | 10% | 375 | 1.6 | 7% | 235 | 1.2 | 9% | 3778 | 3.1 | 10% |
| Salad | 2678 | 3.4 | 8% | 483 | 2.1 | 10% | 723 | 3.6 | 28% | 3883 | 3.2 | 10% |
| Dessert | 0 | 0.0 | 0% | 156 | 0.7 | 3% | 0 | 0.0 | 0% | 156 | 0.1 | 0% |
| Hard grating cheese | 0 | 0.0 | 0% | 10 | 0.0 | 0% | 20 | 0.1 | 1% | 30 | 0.0 | 0% |

Notes: C1, canteen 1; C2, canteen 2; C3, canteen 3; PW, plate waste; Contrib, contribution relative to total plate waste.

| **B.** | **C1** | | | **C2** | | | **C3** | | | **Total** | | |
| --- | --- | --- | --- | --- | --- | --- | --- | --- | --- | --- | --- | --- |
| **Food course category** | **total CF**  **(g CO_2_ eq.)** | **per capita CF (g CO_2_ eq.)** | **Contrib (%)** | **total CF**  **(g CO_2_ eq.)** | **per capita CF (g CO_2_ eq.)** | **Contrib (%)** | **total CF**  **(g CO_2_ eq.)** | **per capita CF (g CO_2_ eq.)** | **Contrib (%)** | **total CF**  **(g CO_2_ eq.)** | **per capita CF (g CO_2_ eq.)** | **Contrib (%)** |
| First course | 9301 | 11.7 | 14% | 1573 | 6.9 | 28% | 261 | 1.3 | 8% | 11135 | 9.1 | 15% |
| Second course | 38642 | 48.4 | 58% | 1078 | 4.7 | 19% | 871 | 4.3 | 27% | 40592 | 33.1 | 54% |
| Side dishes | 10589 | 13.3 | 16% | 1632 | 7.2 | 29% | 1052 | 5.2 | 32% | 13273 | 10.8 | 18% |
| Bread and substitutes | 2765 | 3.5 | 4% | 329 | 1.4 | 6% | 273 | 1.4 | 8% | 3367 | 2.7 | 4% |
| Fruit | 1144 | 1.4 | 2% | 119 | 0.5 | 2% | 105 | 0.5 | 3% | 1368 | 1.1 | 2% |
| Salad | 3818 | 4.8 | 6% | 356 | 1.6 | 6% | 402 | 2.0 | 12% | 4577 | 3.7 | 6% |
| Dessert | 0 | 0.0 | 0% | 419 | 1.8 | 7% | 0 | 0.0 | 0% | 419 | 0.3 | 1% |
| Hard grating cheese | 0 | 0.0 | 0% | 153 | 0.7 | 3% | 307 | 1.5 | 9% | 460 | 0.4 | 1% |

Notes: C1, canteen 1; C2, canteen 2; C3, canteen 3; CF, carbon footprint; Contrib, contribution relative to total carbon footprint of plate waste.

| **C.** | **C1** | | | **C2** | | | **C3** | | | **Total** | | |
| --- | --- | --- | --- | --- | --- | --- | --- | --- | --- | --- | --- | --- |
| **Food course category** | **total WF**  **(L H_2_O)** | **per capita WF (L H_2_O)** | **Contrib (%)** | **total WF**  **(L H_2_O)** | **per capita WF (L H_2_O)** | **Contrib (%)** | **total WF**  **(L H_2_O)** | **per capita WF (L H_2_O)** | **Contrib (%)** | **total WF**  **(L H_2_O)** | **per capita WF (L H_2_O)** | **Contrib (%)** |
| First course | 8017 | 10.0 | 14% | 1731 | 7.6 | 28% | 238 | 1.2 | 7% | 9986 | 8.1 | 15% |
| Second course | 28452 | 35.7 | 50% | 893 | 3.9 | 14% | 719 | 3.6 | 22% | 30064 | 24.5 | 46% |
| Side dishes | 10474 | 13.1 | 19% | 2150 | 9.4 | 34% | 1134 | 5.6 | 35% | 13758 | 11.2 | 21% |
| Bread and substitutes | 3047 | 3.8 | 5% | 363 | 1.6 | 6% | 302 | 1.5 | 9% | 3712 | 3.0 | 6% |
| Fruit | 4257 | 5.3 | 8% | 265 | 1.2 | 4% | 149 | 0.7 | 5% | 4671 | 3.8 | 7% |
| Salad | 2220 | 2.8 | 4% | 526 | 2.3 | 8% | 590 | 2.9 | 18% | 3337 | 2.7 | 5% |
| Dessert | 0 | 0.0 | 0% | 260 | 1.1 | 4% | 0 | 0.0 | 0% | 260 | 0.2 | 0% |
| Hard grating cheese | 0 | 0.0 | 0% | 53 | 0.2 | 1% | 105 | 0.5 | 3% | 158 | 0.1 | 0% |

Notes: C1, canteen 1; C2, canteen 2; C3, canteen 3; WF, water footprint; Contrib, contribution relative to total water footprint of plate waste.

**Supplementary Table 3.** Comparison of plate waste in grams, in terms of energy, macronutrient composition, carbon footprint and water footprint by sex and age group in the total sample of meals that were not fully consumed (each participant was considered only once; N=184).

| **Variable** | **Age group I**  **(N=66)** | **Age group II**  **(N=86)** | **Age group III**  **(N=32)** | **p-value^1^** | **Female**  **(N=109)** | **Male**  **(N=75)** | **p-value^2^** |
| --- | --- | --- | --- | --- | --- | --- | --- |
|  | **Median (IQR)** | | |  | **Median (IQR)** | |  |
| PW (g) | 97.5 (49.1-178.8) | 68.4 (35.4-121.7) | 50.0 (38.4-130.6) | 0.0710 | 84.6 (46.2-168.2) | 50.0 (25.0-100.0) | **0.0023** |
| PWE (kcal) | 105 (44.3-201) | 85 (37-148) | 65 (38-124) | 0.2482 | 95 (44-189) | 69 (37-119) | **0.0422** |
| Protein (g) | 4.0 (1.9-9.0) | 3.4 (1.1-6.2) | 2.1 (1.1-4.4) | 0.0824 | 4.3 (1.7-9.0) | 2.2 (1.1-4.3) | **0.0013** |
| Lipids (g) | 3.5 (1.7-7.4) | 2.5 (0.6-6.5) | 2.7 (1.3-4.1) | 0.2642 | 3.7 (1.8-7.5) | 1.8 (0.2-5.0) | **<0.0001** |
| Carbohydrates (g) | 13.3 (2.2-20.4) | 8.4 (2.6-24.6) | 7.6 (3.1-16.3) | 0.8077 | 8.1 (2.2-24.6) | 8.4 (3.9-18.6) | 0.8371 |
| Fiber (g) | 1.6 (0.8-3.3) | 1.1 (0.5-2.0) | 1.3 (0.7-2.0) | 0.1585 | 1.6 (0.8-2.8) | 1.1 (0.5-2.0) | 0.0705 |
| CF (g CO_2_ eq.) | 105.5 (33.7-271.1) | 67.8 (25.3-184.8) | 56.2 (28.1-115.3) | 0.0802 | 116.4 (47.3-268.9) | 53.2 (20.5-117.9) | **<0.0001** |
| WF (L H_2_O) | 148.9 (53.0-207.7) ^†^ | 76.5 (38.7-180.0) | 60.7 (33.8-126.6) ^†^ | **0.0306** | 146.8 (54.3-207.7) | 53.0 (32.7-144.2) | **<0.0001** |

Notes: PW, plate waste; CF, carbon footprint; WF, water footprint; Age group I, ≤34 years old; Age group II, 35-64 years old; Age group III, ≥65 years old. ^1^ Kruskal-Wallis test; ^2^ Wilcoxon test. ^†^ Significantly different according to the Dunn test, p=0.0343.
